# Jumping vs. running: Effects of exercise modality on aerobic capacity and neuromuscular performance after a six-week high-intensity interval training

**DOI:** 10.1371/journal.pone.0281737

**Published:** 2023-02-10

**Authors:** María Venegas-Carro, Joshua T. Herring, Simon Riehle, Andreas Kramer

**Affiliations:** Department of Sport Science, University of Konstanz, Konstanz, Germany; University of Hartford College of Education Nursing and Health Professions, UNITED STATES

## Abstract

**Purpose:**

High-intensity interval training (HIIT) has proven to be effective in improving endurance capacity and muscle endurance. However, its potential to improve other aspects of physical performance such as strength and power has not been well explored, and most research studies used only running and cycling as exercise modalities. Here, we compared the effects of jumping versus running as exercise modalities during a 6-week HIIT.

**Methods:**

46 participants (24±3 years, 171±9 cm, 68±13 kg, 22 women) were randomly allocated to one of three groups: countermovement jump training, running training, or control. The two training groups underwent a 6-week HIIT with 3 training sessions per week. Both training protocols had identical training frequency, number of series and work/rest durations (on average 7 series of 25s, with a rest of 25s between series). Before and after the training period, aerobic capacity and neuromuscular performance were assessed.

**Results:**

Analyses of variance revealed a significant group*time interaction effect for maximal aerobic capacity (p = 0.004), and post hoc analyses showed a significant increase in the running group (p < .001, +7.6%). Analyses of the maximal voluntary contraction revealed only a significant increase in the jumping group (plantar flexion +12.8%, knee extension +8.2%). No interaction effects were found for maximal power or jump height.

**Conclusion:**

Despite identical programming, the choice of exercise mode profoundly affected the training adaptations: the running group significantly increased aerobic capacity, and the jump group significantly increased leg strength. These results underline the importance of exercise modality in physical performance adaptations.

## Introduction

High-intensity interval training (HIIT) is a type of training that consists of repeated bouts of exercise with near-maximal effort (an intensity of at least 80% of the maximal heart rate), separated by short periods of recovery [1]. Several studies have found this type of training to be at least as effective at improving aerobic capacity and muscle endurance as moderate-intensity continuous training (MICT), with the additional advantage that it also requires less volume and time commitment [2, 3]. However, most studies comparing HIIT to MICT have mainly used exercise modalities such as running or cycling [4–9]. In addition, the focus of most HIIT studies was the cardiorespiratory fitness (CRF) improvement [10], usually disregarding other aspects of physical performance such as strength and power [11]. Therefore, the potential of HIIT to do so has not been well explored, with only a few studies employing other exercise modalities such as bodyweight exercises or plyometric training.

Plyometric training has been extensively used as part of the training program for different sports, not only those that require jumping but also sprinting [12], and its potential to increase maximal strength, maximal rate of force development and power has been well-documented [12, 13]. The study by Potteiger and co-workers tested how 8 weeks of only plyometric training or a combination of plyometric and aerobic exercise could improve leg muscle power and produce changes in muscle fibre characteristics [14]. Their results revealed that the plyometric training alone was able to increase not only peak power output and muscle fibre size, but also maximal oxygen uptake, as much as the combination of this type of training with aerobic exercise. According to these results, it is tempting to assume that when used as a HIIT exercise modality, one could reap the benefits in muscle strength and power produced by plyometric training, and additionally get the improvement in aerobic capacity that is usually associated with HIIT. Indeed, the results of a recent bedrest study showed that a high-intensity jump training, consisting of countermovement jumps (CMJ) and reactive hops, was able to maintain CRF, neuromuscular performance and bone strength, whereas the control group markedly deteriorated in all of those areas [15–17]. Additionally, a cross-sectional study showed that a training session consisting of solely CMJs can elevate the heart rate to near maximal levels and increase oxygen consumption to almost maximal intensities if the rest duration between jumps and between series of jumps is sufficiently short [18].

Therefore, the present study aimed to evaluate whether a high-intensity jump training can not only be used to maintain aerobic capacity and neuromuscular performance during prolonged periods of inactivity, but also to increase physical performance parameters in an otherwise normally active population in comparison to a running HIIT, which is next to cycling the preferred and most used exercise modality in studies comparing HIIT to MICT [4–9]. We hypothesized (1) that a 6-week high-intensity jump training would improve aerobic capacity as much as a 6-week HIIT using the same training frequency, number of series and work/rest durations but running as an exercise modality, and (2) that the jump training group would improve leg strength and power more than the running group.

## Methods

### Study design

**Fig 1:**
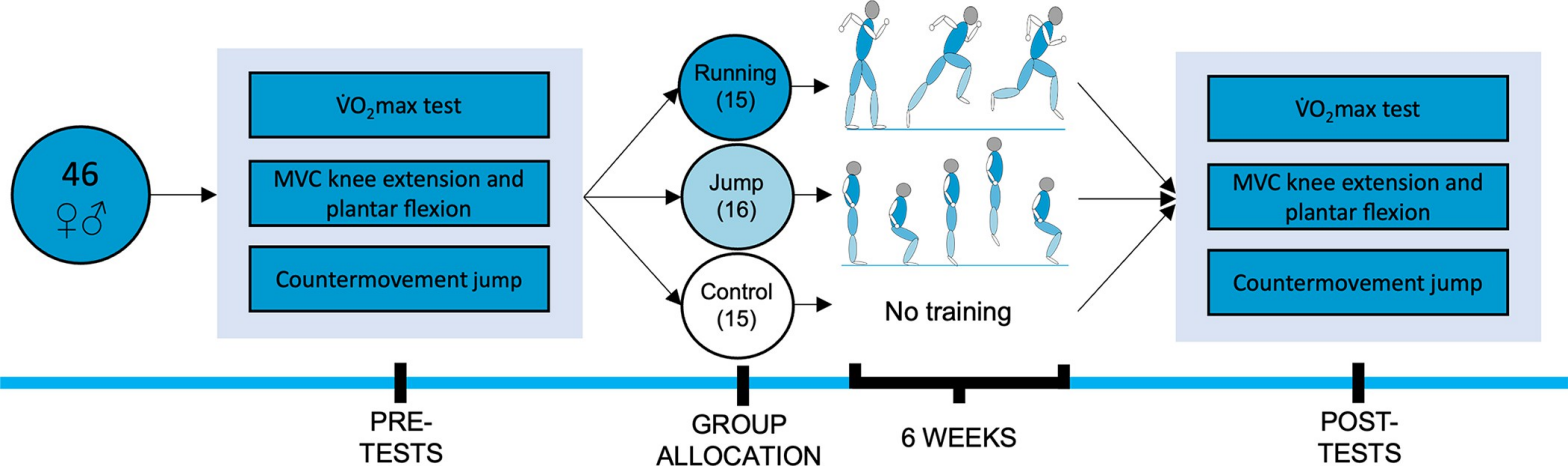
Study design.

This randomized controlled training study started with one set of baseline measurements, followed by 6 weeks of training for the jumping (JT) and running (RT) groups, or no changes to their daily routine for the control group (CON). Post-tests were performed 48 hours after the last training for the training groups [19, 20], and 6 weeks after the baseline measurements for CON. For a study design overview see Fig 1. After the initial tests, participants were randomly assigned to one of the 3 groups using a random number generator (www.randomize.org). The primary outcome concerning the efficacy of the training interventions was maximal aerobic capacity during a bicycle ramp test. Secondary outcomes were maximal voluntary contraction (MVC) of the leg muscles during isometric knee extension (KE) and plantar flexion (PF) and jump performance during a CMJ.

After the pre-tests, participants were randomly allocated to one of the three groups (control, running, jump). Participants in the training groups were subjected to either running training or jump training three times per week for 6 weeks. Participants in the control group were subjected to no intervention for 6 weeks. After the 6 weeks, all participants completed the same tests as at the beginning of the study (post-tests). Pre and post-tests included countermovement jumps, maximal aerobic capacity ($\dot{V}O_{2\max}$), and maximal voluntary contraction (MVC) during knee extension and plantar flexion.

### Subjects

A total of 52 participants were recruited. Subjects were recreationally active (average weekly activity of 2.8 ±2.7hr). Exclusion criteria involved a relative oxygen uptake ($\dot{V}O_{2\max}$) higher than 55 mL/min/kg for men and 49.5 mL/min/ kg for women to exclude well-trained subjects. Participation in the study was voluntary, and the study protocol was in accordance with the Declaration of Helsinki and approved by the Ethics Committee of the University of Konstanz (Year: 2018/ Title: “A comparison of the effects of high-intensity interval jump training and sprint interval training on physical performance”). Before any involvement in the study, participants were informed of study details and associated potential risks. They filled out the Physical Activity Readiness Questionnaire for Everyone (PAR-Q+) [21] to ensure that no contraindications to sports participation were present. Afterwards, they signed the written informed consent form. A total of 6 participants dropped out during the training period (reasons: 2 fell ill and were not able to train, 2 got injured outside of the study, 1 lacked time to train, and 1 decided to no longer participate). Their data were excluded from further analyses. Group characteristics at baseline of the remaining 46 participants are detailed in Table 1.

**Table 1:** Group characteristics at baseline.

	Group			Total	*p*-value
	Running	Jump	Control		
N	15	16	15	46	
Females	5	11	6	22	0.058
Age [yrs]	23.3±2.9	24.5±3.6	24.8±3.1	24.2±3.2	0.423
Height [cm]	173.0±8.1	169.2±10.1	172.3±9.1	171.5±9.1	0.475
Weight [kg]	67.7±10.0	65.2±13.2	71.5±15.1	68.0±12.9	0.41
BMI [kg/m^2^]	22.5±1.8	22.7±3.2	23.8±3.3	23.0±2.8	0.386
Body fat [%]	13±5	19±7	17±7	16±7	0.040*

Mean±SD group characteristics at baseline separated by group. Body fat percentage was determined using the 7-site skinfold assessment test. The last column on the right shows group differences at baseline, evaluated via a one-way analysis of variance.

An * symbol denotes significant *p-*values (*p*<0.05).

### Anthropometric assessments

Height and weight were measured with a stadiometer and a weight scale (Seca GmbH, Hamburg, Germany). The 7-site skinfold thickness measurements (chest, sub scapula, mid axilla, triceps, abdomen, supra iliac, and thigh) were taken with a calliper (Harpenden Skinfold Caliper, Baty International, Burgess Hill, United Kingdom) to estimate body fat percentage with the seven-site equation [22, 23].

### Isometric muscle strength

**Fig 2:**
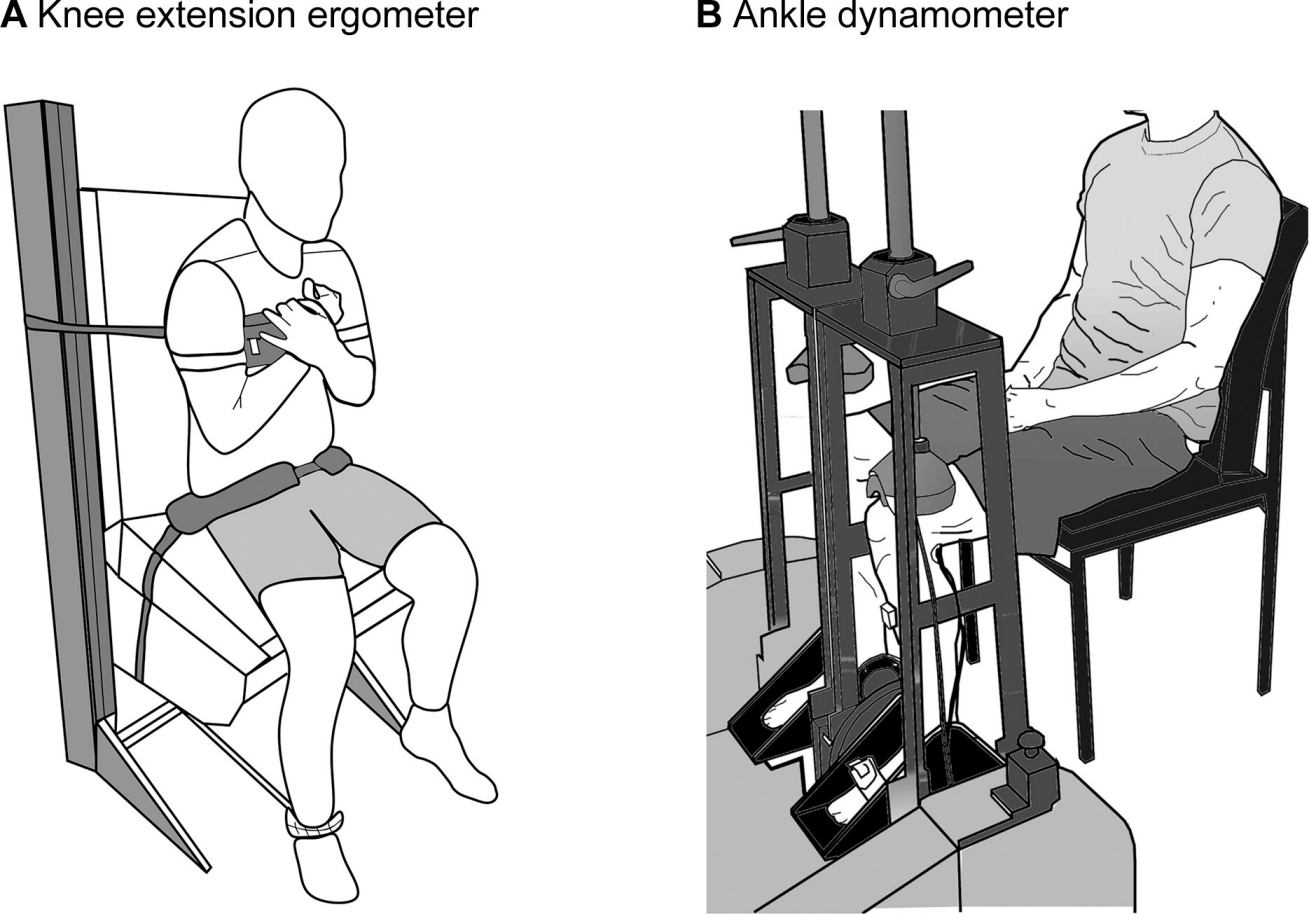
Ergometer and dynamometer used to test isometric leg strength. (A) Custom-made ergometer for the isometric maximal voluntary contraction during knee extension. The participant’s left ankle was attached to the ergometer’s lever arm about 3 cm above the lateral malleolus. Adjustable straps and pads on the chest and hip were used to minimize extraneous body movements. (B) Custom-made ankle dynamometer for the isometric maximal voluntary contraction during plantar flexion. The participant’s foot was fixed to the bottom plate with a strap to prevent plantar extension. The position of the knee was fixed with a clamp to prevent the lifting of the heel. Figure adapted with permission from Kümmel et al., [24].

Before any test was done, participants performed a warm-up that consisted of 3-minute cycling on the cycle ergometer at 30 W, 3 bodyweight squats, 3 submaximal CMJs, and 10 submaximal hops. The MVC was then assessed with a custom-made knee ergometer and ankle dynamometer for KE (Fig 2A) and PF (Fig 2B) of the left leg. Performance in both movements was recorded in the sitting position (KE: knee joint angle of 90°, hip angle of 60°; PF: 90° for hip, knee, and ankle joints). For both movements, the test consisted of 5–6 submaximal familiarization contractions with increasing intensity, separated by 30 s each, followed by 3 MVC with a duration of approximately 3 s. Between each MVC, participants rested for 90s. The instruction was to “push quickly as hard as you can”, and strong verbal encouragement was provided during every trial.

Force (KE, analogue signals of the ergometer load cells) and torque data (PF) were amplified (Kistler 5006 and 5015, Kistler Group, Winterthur, Switzerland), sampled at a frequency of 1000 Hz (CED Power1401, CED Ltd., Milton, UK) and recorded and stored for further analyses with MATLAB (version R2020b, MathWorks Inc., Massachusetts, USA). The analyses consisted of correcting potential offsets and applying a 6th-order zero-lag Butterworth filter (cut-off frequency 15 Hz). For KE, torque was calculated for every participant as the product of force (N) times the perpendicular distance (m) between the lateral epicondyle of the left knee to the lever arm of the ergometer. The peak torque (N⋅m) of every trial for both KE and PF was extracted, and the highest of the three trials was kept for further analysis.

### Jumping power

To assess maximal lower extremity power, three CMJs (with hands akimbo) with a 1-min rest between jumps were performed on a force plate (Leonardo Mechanograph GRFP, Novotec Medical GmbH, Pforzheim, Germany). The participants were instructed to quickly drop to a squat position and immediately jump as high as possible. Ground reaction forces were sampled and recorded at 800 Hz. Data acquisition and analysis were performed with Leonardo Mechanography software (version 4.3b01.93, Novotec Medical GmbH, Pforzheim, Germany). Maximal jump height (JH) and jump power were extracted and only the highest value was retained for further analysis.

### Aerobic capacity

The protocol to determine maximal aerobic capacity consisted of the following phases: (1) a 30s phase of seated rest on the ergometer for baseline measurements; (2) a 3-min warm-up at double the set increment of the ramp test (e.g., if the ramp was set to 15 W per min, the warm-up was performed at 30 W); (3) an 8-12-min ramp test [25] with increments ranging from 10 to 30 W per minute. The ramp protocol for each participant was chosen based on the amount of exercise they indicated to perform regularly. The participants were asked to keep a cadence ranging from 80 to 100 (previously chosen by the subject on the warm-up) during the whole test until volitional exhaustion was reached despite strong verbal encouragement. Finally, (4) a 5-min recovery phase, where participants sat on the cycle ergometer without pedalling. Criteria to determine that a maximum effort was achieved were a respiratory exchange ratio (RER) of at least 1.10 [26] or a rate of perceived exertion (RPE) ≥18 [27]. If none of these criteria was met, the test was excluded from the analyses, which was the case for one participant.

All breath-by-breath oxygen uptake ($\dot{V}O_{2}$) and carbon dioxide ($\dot{V}CO_{2}$) emissions were recorded with the ergospirometer Ergostik (Geratherm Respiratory GmbH, Bad Kissingen, Germany). The raw data were extracted with the Blue Cherry program (version 1.2.2.7, Geratherm Respiratory GmbH, Bad Kissingen, Germany) and the heart rate was monitored with a wireless heart rate belt (Polar Wearlink, Polar Electro Oy, Kempele, Finland). The spirometry data of the ramp test was filtered in MATLAB by first applying a 5-breath median and then a 30-s moving average [28]. Afterwards, the following peak values were used for further analyses: absolute and relative $\dot{V}O_{2}$ ($\dot{V}O_{2\max}$), maximal heart rate (HR), and maximal ergometer power.

### Training

The two training groups (RT and JT) performed a total of 18 sessions over the course of the six-week training phase (3 training sessions per week, with at least 48 hours of rest between sessions). An overview of all sessions is given in Table 2. The training frequency and the number of series, as well as the work and rest durations, were the same for both training groups to ensure a similar exercise prescription [27, 29]. The first few sessions were meant to familiarize the participants with the demands of the HIIT sessions, which is why they were shorter and had longer rest durations. The variations used in the following sessions were based on the work by Kramer *et al*., [18], and were meant to keep the training interesting and thus compliance high. Total session time varied from 4 min 20 s to 7 min 30 s, and total work time per session from 1 min 40 s to 4 min in both groups. The warm-up for every training session consisted of (1) a 3-min movement preparation phase (individual mobilisation and stretching exercises), (2) 3 min of self-paced jogging, and (3) a series of standardized jump (JT) or running prep (RT) exercises. Before the first session, participants were introduced to the correct warm-up and training exercises. Immediately after every training session, the RPE on a 6–20 Borg Scale [30] was recorded for every participant.

**Table 2:** Detailed parameters of the training sessions.

Week	Training number	Number of series	Work duration [s]	Rest duration [s]
1	1	6	20	40
	2	8	20	40
	3	6	20	30
2	4	6	25	30
	**5**	**7**	**25**	**30**
	6	6	30	30
3	**7**	**7**	**30**	**30**
	8	6	30	20
	9	7	30	20
4	**10**	**7**	**30**	**20**
	11	8	30	30
	**12**	**4**	**30**	**15**
5	13	7	30	20
	14	6	20	10
	15	8	30	30
6	**16**	**7**	**30**	**20**
	**17**	**7**	**20**	**10**
	18	5	20	40

Number of series, work duration and rest duration of the 18 training sessions. The two training groups did not differ with respect to these characteristics, only the exercise modality was different (jump vs. running). For six training sessions (marked in bold), the jump technique was changed (continuous jumps, resulting in a more intensive training).

### Jump training

The exercise modality used for this group was CMJs. Participants were encouraged throughout the entire session to jump as high as possible and to exert maximal effort during every repetition. Of the 18 total sessions, 12 used CMJs with almost no rest between jumps (only enough time to reposition, legs extended), and six sessions consisted of continuous jumps, meaning that there was no rest between the jumps, so that participants were required to use the landing position with bent legs as a starting position for the next jump. Participants were strongly encouraged to always jump as high as possible and to exert maximal effort on every trial.

### Sprint training

Participants in the RT group used running as the modality of exercise. The running sprints were completed along the width of a basketball court (15 m), stopping on the last two meters, and reaccelerating in the other direction as fast as possible. Participants were strongly encouraged to always run as fast as possible and to exert maximal effort on every trial.

### Statistics

Changes as a result of the training were analysed with a two-way-factorial-mixed-repeated-measures analyses of variance (rmANOVA) with group (3 levels, Jump vs. Running vs. Control) as between-subjects factor and time (2 levels, Pre vs Post) as the repeated measure for each of the 8 dependent variables (relative and absolute $\dot{V}O_{2\max}$, peak power, maximal HR, MVC KE, MVC PF, CMJ height, CMJ power). In case of significant interaction effects, the rmANOVAs were followed up with Bonferroni-corrected post hoc tests (family of three, paired *t*-tests for the pre-post within-group comparisons). RPE after each training session was analysed using a two-way-factorial-mixed-rmANOVA with group (2 levels, Jump vs. Running) as the between-subjects factor, and time (18 levels, the 18 training sessions) as the repeated measure. Independent-samples *t*-tests were used to evaluate differences between individual sessions (Jump vs. Running). The differences in age, height, weight, BMI, and body fat percentage between groups at the beginning of the study were evaluated using one-way ANOVAs (Jump vs. Running vs. Control). Analyses were executed with JASP (version 0.14.1, University of Amsterdam, Netherlands). Group data are presented as means ± standard deviations. The significance level was set to 0.05. Sample size estimation was calculated with G*Power 3, version 3.1.9.6 [31] (effect size of 0.5 –based on the findings of Saez de Villareal and colleagues [32]–a power of 0.8, and an alpha error of 0.05, resulting in an estimated total sample size of 42 participants). All figures were created in the statistical environment R version 4.1.0 [33] with the use of the packages *tidyverse* [34], *ggplot2* [35] and *ggsignif* [36].

## Results

### Aerobic exercise capacity

**Fig 3:**
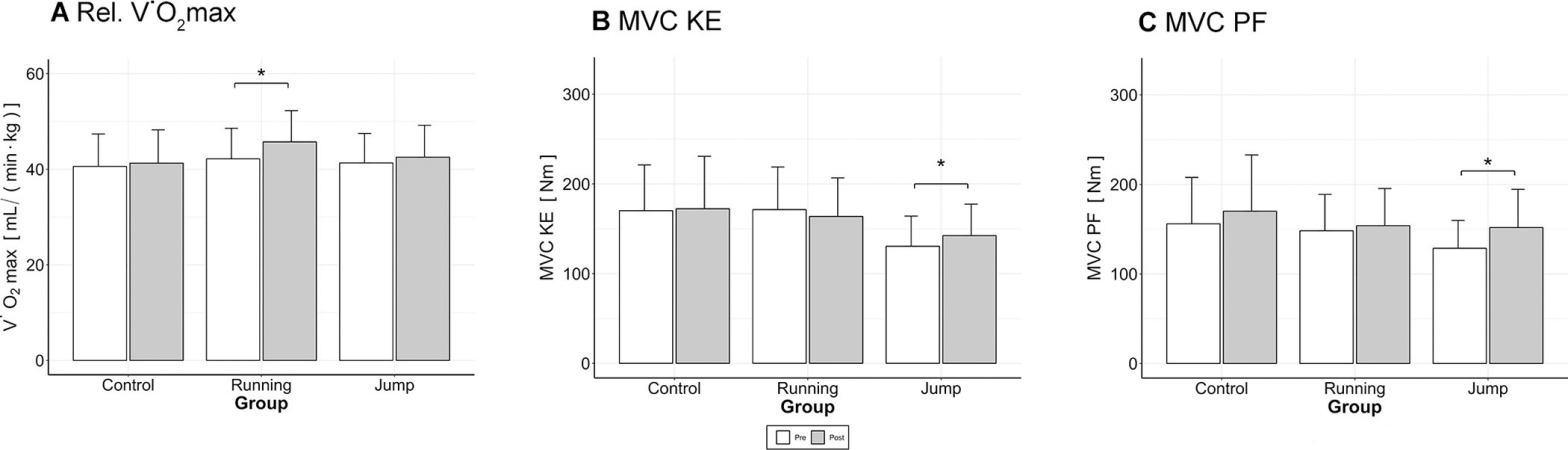
Changes in aerobic capacity as well as lower extremity strength. Results of the pre-tests in white, and post-tests in grey; data refer to group mean and SD. (A) relative $\dot{V}O_{2\max}$, (B) Maximal voluntary contraction (MVC) during knee extension (KE), (C) MVC during plantar flexion (PF). The * symbols denote significant differences between pre-and post-values (Bonferroni-corrected post hoc tests).

Statistical analyses revealed a statistically significant group*time interaction effect for the relative $\dot{V}O_{2\max}$ (*p* = 0.004) and the absolute $\dot{V}O_{2\max}$ (*p*<0.001) (Table 3). Subsequent post hoc analyses revealed a significant increase in RT for both relative and absolute $\dot{V}O_{2\max}$ (*p*<0.001) (Fig 3A). In the JT group, the follow-up tests were significant for the absolute $\dot{V}O_{2\max}$ (*p* = 0.033) but not for the relative $\dot{V}O_{2\max}$ (*p* = 0.186). For the CON group, the post hoc tests for the absolute and the relative $\dot{V}O_{2\max}$ were not significant (*p* = 0.483 and *p =* 0.522, respectively). There was a significant effect of group*time for the peak power during the incremental ramp test (*p* = 0.004) (Table 3) and follow-up tests revealed a significant increase for both training groups (RT, *p*<0.001; JT, *p*<0.001) and the CON group (*p* = 0.006). No statistical difference was found for the peak HR during the ramp test (Table 3).

**Table 3:** Aerobic exercise capacity, leg strength, and jump performance.

	Running		Jump		Control			Group* time	Time
	Pre	Post	Pre	Post	Pre		Post		
Spiroergometry									
rel. $\dot{V}O2_{\max}$ [mL/(min kg)]	42.2 ±6.4	45.7 ±6.5	41.3 ±6.2	42.5 ±6.7	40.6 ±6.8		41.3 ±7.0	0.004*	< .001*
	+7.6%		+2.6%		+1.7%				
abs. $\dot{V}O2_{\max}$ [L/min]	2.90 ±0.8	3.18 ±0.8	2.68 ±0.7	2.8 ±0.7	2.94 ±0.9		3.0 ±0.9	< .001*	< .001*
	+9.0%		+4.1%		+1.9%				
Peak power [W]	252 ±67	278 ±70	234 ±57	248 ±60	254 ±76		265 ±80	0.004*	< .001*
	+9.3%		+5.9%		+4.0%				
max. HR [beats/min]	187 ±10	187 ±8	189 ±8	189 ±7	189 ±9		190 ±8	0.586	0.972
	-0.2%		-0.1%		+0.4%				
Isometric strength									
MVC KE [N⋅m]	171.4 ±47.5	163.8 ±42.9	130.5 ±33.6	142.4 ±35.2	170.2 ±51.0	172.3 ±58.5		0.007*	< .001*
	-4.7%		+8.2%		-1.5%				
MVC PF [N⋅m]	148.2 ±40.6	153.9 ±41.5	128.7 ±31.2	151.8 ±42.7	156 ±51.8	170.1 ±62.8		0.006*	0.024*
	+2.5%		+12.8%		+4.3%				
Peak power									
CMJ height [cm]	44.0 ±10.7	43.8 ±10.0	39.6 ±8.0	39.2 ±7.5	45.1 ±9.6	45.5 ±9.3		0.726	0.89
	-0.6%		-0.8%		+0.7%				
CMJ power [N]	49.2 ±12.0	47.2 ±9.8	43.4 ±7.8	42.4 ±7.9	49.4 ±10.1	48.4 ±10.5		0.723	0.035*
	-3.8%		-2.5%		-2.6%				

Values expressed as mean ± SD, separately for the Control, Running, and Jump group, before (pre) and after (post) the 6 weeks. Percent values reflect the average increase or decrease at the post-measurements compared to pre-tests. The last columns on the right contain the group*time interactions and the main effect of time, respectively.

* Denotes significant *p-*values (*p*<0.05). rel. = relative, abs. = absolute, max. = maximal, HR = heart rate, maximal voluntary contraction = MVC, KE = knee extension, PF = plantar flexion, CMJ = countermovement jump.

### Maximal strength

The results of the MVC tests showed a significant group*time interaction effect for both PF (*p* = 0.006) and KE (*p* = 0.007) (Table 3), and the post hoc tests showed a significant increase for JT only (*p* = 0.018 for PF, and *p*<0.001 for KE), not for RT (*p* = 1.0 for PF and *p =* 0.330 for KE) or CON (*p* = 0.168 for PF and *p =* 1.0 for KE) (Fig 3B and 3C).

### Peak power

CMJ power analyses showed no significant group*time interaction effect (*p* = 0.72), only a significant main effect of time (*p* = 0.035) (Table 3). CMJ height showed no significant group*time interaction effect or main effect of time (Table 3).

### Training sessions

**Fig 4:**
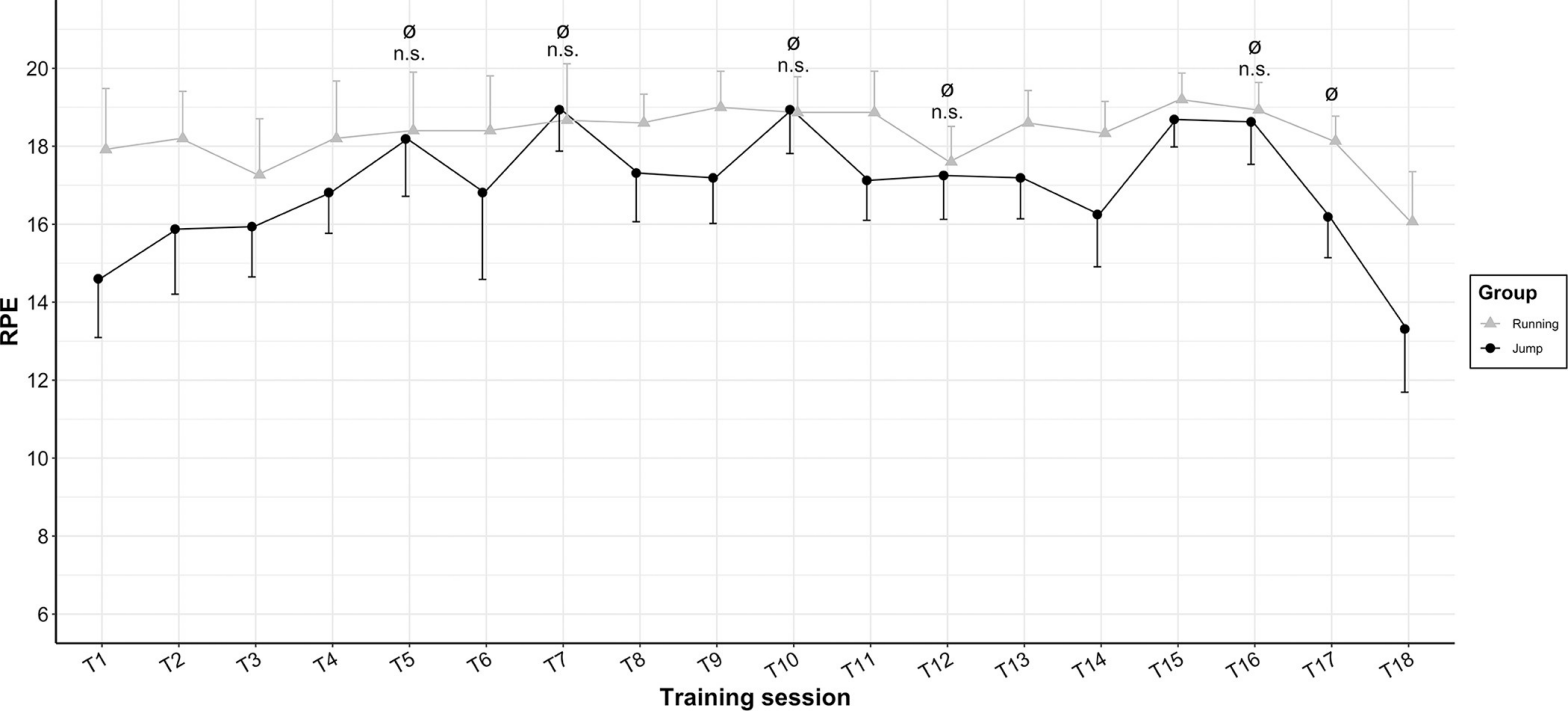
Average rated perceived exertion (RPE, mean and SD) for the two training groups during the 18 training sessions. Data for the jump training group is shown in black, and data for the running training group is in grey. Ø = on top of the training session denotes the jump training sessions with continuous jumps. n.s. = denotes training sessions with no significant differences in RPE between JT and RT (evaluated with independent-samples *t-*tests).

All subjects in the intervention groups completed the 18 training sessions as scheduled. On average, the training protocol consisted of 7 series per session with a work duration of 25s and a rest duration of 25s. The RPE was assessed at the end of all 18 sessions, showing an overall average of 18 ± 0.7 for RT and 17 ± 0.8 for JT (*p*<0.001 for the group*time interaction effect). When looking at the training sessions separately with independent-samples *t*-tests, only five averaged-RPE results were not significantly different between intervention groups (training sessions 5, 7, 10, 12, and 16), and all these five training sessions were the ones performed with continuous jumps in the JT group (Fig 4).

## Discussion

This randomized controlled trial aimed to compare the effects of two HIIT protocols using either jumping or running as an exercise modality. The study’s main findings were that only the running HIIT group significantly increased their relative aerobic capacity ($\dot{V}O_{2\max}$) after the six-week training period, and the jump HIIT group significantly increased maximal leg strength, with no improvements in maximal power in either group. As the two training programs were identical in training frequency, number of series, work intervals and rest intervals, these results underline the importance of exercise modality. In addition, the differences in RPE between the two slightly different jump training sessions suggest that not only exercise modality but also subtle differences in how the exercise is performed can play a role.

### Aerobic capacity

The significant effect of RT on aerobic capacity is in line with the results of previous HIIT studies using different exercise modalities [4]. This increase of about 7.6% in $\dot{V}O_{2\max}$ is also comparable to what HIIT studies that used running as an exercise modality and similar duration found. For example, Litleskare and colleagues reported an increase in $\dot{V}O_{2\max}$ of 6% after eight weeks of training with 30s work intervals [5], MacPherson et al. found an improvement of 11.5% after 6 weeks of sprint training with 60s work intervals [6], Nalçakan and co-workers reported a 10% increase after six weeks of running HIIT with 20s work intervals [37], Flensted-Jensen and colleagues an improvement of 7% after 6 weeks of HIIT cycling with 60s work intervals [38], and Arazi et al. an increase of 7% after six weeks of running HIIT with 90s work intervals [39].

However, the lack of statistically significant improvement in relative $\dot{V}O_{2\max}$ that we observed in our study after jump HIIT was surprising given the positive results of previous studies using jumps and other types of bodyweight exercises. For instance, Menz and colleagues [40] compared four weeks of running HIIT to functional HIIT (20s work duration, functional exercises: various types of jumps, squats, push-ups, and mountain climbers) and found similar increases in $\dot{V}O_{2\max}$ in both groups (+13% in the running HIIT group vs. +11% in the functional HIIT group). Even though the groups consisted of only 8 participants each and there was no control group, these results indicate similar effects of other exercise modalities compared to running. Moreover, Schaun et al. compared the efficacy of body-weight exercises to both a traditional HIIT protocol and an endurance training on a treadmill [41]. No matter the important differences in exercise modality and volume, they found that all groups were able to increase $\dot{V}O_{2\max}$ significantly. Similarly, McRae and colleagues compared a four-week endurance training to HIIT using squat thrusts, burpees, mountain climbers and other whole-body exercises [42]. Their results showed a significant 8% increase in peak $\dot{V}O_{2}$ in the HIIT group, versus 7% in the endurance group. This improvement was also accompanied by a significant increase in muscle endurance in the HIIT group. Finally, the results of a longer study by Lu and co-workers suggest that functional HIIT can increase $\dot{V}O_{2\max}$, but less so than running HIIT: the authors compared 12 weeks of running HIIT to functional HIIT (20s work duration, functional exercises: squats, jumping jacks, burpees, mountain climbers, side-to-side squats), resulting in $\dot{V}O_{2\max}$ improvements of 17% for the running group and 12% for the functional HIIT group [43].

One reason that could explain the lower improvement in aerobic capacity in JT in our study compared to the aforementioned studies, is that their training consisted of a series of different types of exercises, and ours of only one, countermovement jumps. It is known that if the same muscle groups are used throughout the whole exercise session, exercise capacity can be limited by blood flow rather than by maximal cardiac output [44]. In addition, muscle strain and fatigue might have also limited the cardiopulmonary demand of the jump training, thus reducing its efficacy to elicit the desired cardiopulmonary adaptations. Another factor to consider is the presumably low time under tension during jumps (particularly for good jumpers with a long flight time), which has been shown to affect the acute neuromuscular response to the training [45].

These potential explanations for the lower improvement in $\dot{V}O_{2\max}$ of JT compared to RT also fit with our observation that the perceived difficulty of the sessions rated by the participants (i.e., RPE) was lower for JT (grand mean: 17 ±0.8) than for RT (grand mean: 18 ±0.7). Although both these numbers are in the recommended range of 15–20 for a HIIT session [29], they were significantly different (*p*<0.001). When subdividing the jump sessions into the twelve sessions with little rest between jumps (only enough time to reposition, legs extended), and the six sessions consisting of continuous jumps (no rest between jumps, using the landing position with bent legs as a starting position for the next jump), it becomes obvious that this difference in RPE is quite substantial when performing the jumps with a bit of rest between jumps, but hardly existent when the jumps are performed without rest between jumps (Fig 4). This is in line with the findings of a previous study by Kramer and colleagues, who examined how differences in rest duration between repetitions and between series affected the acute cardiopulmonary demand and RPE [18]. Their results showed that with short or no rest intervals (especially between repetitions), jump HIIT can elicit near-maximal values of heart rate, $\dot{V}O_{2}$ and RPE. Consequently, our programming of the jump HIIT sessions was probably not optimal: for higher cardiopulmonary demand and longer absolute times above 90% of $\dot{V}O_{2\max}$, more series, longer work intervals, shorter breaks between series, and only training sessions without rest between repetitions would most likely have yielded better effects. We suspected as much when designing the training sessions, but the limiting factor was the running group: it was considered that more series, work interval durations longer than 30s, and shorter breaks between series would be excessive for the RT group, given the “all-out” nature of sprints [46]. However, for jump HIIT, up to 12 series with a work duration of 40s each have already been successfully used [18], and adding series to a training session of lower intensity has been shown to increase the so-called $\dot{V}O_{2}$ slow component [47]. Thus, without the constraint to keep the jump HIIT sessions comparable to the running HIIT sessions, jump HIIT could have been programmed in a more demanding and therefore more effective way.

This goes to show that it is difficult to compare different types of HIIT training, especially when using different types of exercise. Potentially, it might be better to use different amounts of series, work durations and rest durations, and concentrate on something like RPE as a gross overall measure of load for comparison’s sake [48]. In any case, it seems to be clear that programming (high-intensity interval) training is not a puzzle that’s easily solved, but has to take into account the goals of the training, work and recovery durations, the number of intervals, and the overall volume of the session [10, 29], but as we’ve seen also the type of exercise, how the exercise is performed, and whether a single type or several different types of exercise are used in one session.

### Leg strength

When programming a HIIT session, the main focus usually is on the cardiorespiratory adaptations that this form of training can elicit [10, 29]. Nevertheless, the results of our study show how with the appropriate type of training, other aspects of physical performance can also be targeted. Participants in the jump group significantly increased their maximal leg strength by 13% (plantar flexion), and 8% (knee extension), respectively. To our knowledge, our study is the first to show improvements in leg strength after jump HIIT. Only in the study by Kramer and colleagues, in participants confined to bed rest for 60 days, the training group that performed jump HIIT during the bed rest period was able to maintain the neuromuscular function of the leg extensors, as opposed to the control group that lost for example about 40% in KE MVC [15]. Normal (i.e., non-HIIT) plyometric training though has consistently been shown to be associated with improvements in lower limb strength [49].

Given the nature of the training (many repetitions with high intensity), it is possible that other aspects of physical performance were also improved, such as local muscle endurance or lactate tolerance. However, we were limited concerning the number of strenuous tests that we could include, so this remains to be tested in future studies. There are studies though that did include tests of this nature and have found positive results. For instance, McRae et al. assessed leg extension muscular endurance with as many repetitions as possible and reported an increase of 40% after 4 weeks of training [42]. Buckley et al. examined the effects of a 6-week multimodal HIIT (using different types of bodyweight exercises) in comparison to a traditional HIIT with rowing as the exercise modality. The results of the muscle endurance tests revealed a 280% increase in the total number of successful repetitions during a back squat only in the multimodal HIIT group [50]. In addition, HIIT has been shown to improve repeated-sprint ability, potentially by improving the ability to recover between high-intensity efforts [51].

### Jump performance

Contrary to what we expected, jump height and power during a CMJ did not improve after jump HIIT. Non-HIIT plyometric training is usually the method of choice to increase jump height, and to a lesser extent also power [32]. For example, Skurvydas et al. tested a low-intensity 8-week plyometric training consisting only of CMJ in prepubertal boys (30 jumps per session and 20s breaks between jumps) and obtained an increase of 36% in CMJ height after the training [52]. According to meta-analyses, plyometric training is expected to increase vertical jump performance by about 5 to 15% [32], and an average increase of 9% for CMJ height [53].

The reasons why jump height and maximal power during jumps were not increased in our study might be due to the high number of repetitions, and the lack of rest between repetitions. Typically, plyometric training is performed in a well-rested state, with few maximal repetitions and ample time for recovery. For instance, Skurvydas et al. suggested that the high increase in jump height in their study (36%) was partially due to the long rest duration between repetitions (20s), preventing muscle fatigue, which also allowed a high power output during every jump [52]. In the context of our study, there was probably a high amount of muscle fatigue that accumulated over the course of the training session, limiting the power output per jump. Indeed, Kramer et al. have shown that during a jump HIIT session with no rest between repetitions or series, the average jump height is only 35% of the maximal jump height and that increasing the rest durations produces a higher average jump height and power [18]. Finally, another potential explanation for our results might be the use of a single exercise mode. According to a meta-analysis, the combination of different plyometric exercises (e.g., squat jumps, CMJ and drop jumps) might be more effective at improving jump performance [32].

### Limitations

The results and analyses of the present study should be considered with the following limitations in mind. First, the running group’s “all-out” modality limited the possibility of longer work durations and a higher number of intervals per session if the training volume was to be kept equal in both groups. This could have limited the intensity achieved in JT, given that more and longer work intervals would most probably have helped to increase cardiopulmonary demand and thus produce higher CRF improvements. Second, with the use of a portable spirometer and a heart rate monitor, we could have recorded oxygen uptake and heart rate during the running and jumping training sessions to assess the actual cardiopulmonary demand, instead of having to rely on the overall measure of RPE. Third, the significant difference in the female/male ratio as well as in body fat percentage between the groups at the beginning of the study is the result of the randomization process and might have had an effect on the results of the study in case men and women responded differently to this kind of training. Finally, the inclusion of a muscle endurance test and a repeated sprint test would have provided further insight into other training effects specific to the two training modalities.

## Conclusion

The results of our study suggest that despite identical programming (equal training frequency, number of series, and work/rest durations), the choice of exercise mode can profoundly affect the training adaptations: RT significantly increased aerobic capacity, and JT significantly increased maximal leg strength. In addition, the differences in RPE between the two slightly different jump training sessions suggest that not only exercise modality, but also subtle differences in how the exercise is performed determine the overall demand of the training. From an applied perspective, running seems to be the more efficient choice as a HIIT exercise modality if the main goal is to elicit improvements in $\dot{V}O_{2\max}$, even though jump HIIT might be able to elicit a broader range of adaptations with optimal programming.
